# Quantifying the performance of dual-use rainwater harvesting systems

**DOI:** 10.1016/j.wroa.2020.100081

**Published:** 2020-12-13

**Authors:** Ruth Quinn, Charles Rougé, Virginia Stovin

**Affiliations:** Department of Civil and Structural Engineering, The University of Sheffield, UK

**Keywords:** Rainwater harvesting tanks, Stormwater management, Water supply, Peak runoff, Performance metrics, Multi-criteria visualisation

## Abstract

Rainwater harvesting systems in urban settings are increasingly relied upon to mitigate pluvial flooding on top of providing an additional water supply. Alternative designs have been proposed to support their dual use. Stormwater management performance is typically evaluated through long-term averages. However, long-term assessment is not aligned with the goal of attenuating the impacts of short duration high-intensity rainfall events. This paper contributes a framework for evaluating the dual-use performance of design alternatives. The framework incorporates a set of stormwater management metrics that provides a robust characterisation of performance during significant rainfall events. To the usual long-term volumetric retention metric, we add: 1) metrics that represent the total volume and duration above predevelopment (greenfield) runoff rates; and 2) robust peak outflow rate and retention efficiencies based on the long-term median of a representative sample of significant rainfall events. Our multi-criteria performance visualisations of alternative dual-use designs highlight the importance of carefully designing the forecast-based controlled release mechanisms built into active systems. This work has direct implications for design guidance standards, which we discuss.

## Highlights

• Existing RWH performance metrics ignore storm event-based runoff quantities.
• We propose a framework comprising seven performance metrics.
• Stormwater control metrics address routine and significant event performance.
• The sensitivity of performance metrics to demand is configuration specific.
• A flow duration curve may provide a suitable basis for regulatory standards.

## Graphical abstract

**Figure:**
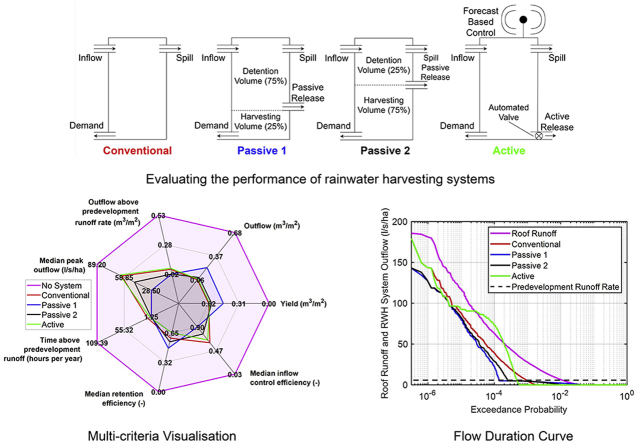

## Abbreviations

C: Controlled outflow (m^3^/5 min)
Cd: Coefficient of discharge (−)
D: Demand (m^3^/5 min)
d: Outlet diameter (m)
E_R_: Retention efficiency (−)
E_CQ_: The proportion of inflow controlled to predevelopment runoff rate (−)
E_ws_: Water supply efficiency (−)
h: Head acting over the centreline of the orifice (m)
QC: Outflow volume controlled above the predevelopment runoff rate (m^3^)
g: Acceleration due to gravity (9.81 m/s^2^)
I: Tank inflow (m^3^/5 min)
N: Number of timesteps where the outflow is above predevelopment runoff (−)
Q: Tank outflow (m^3^/5 min)
Q_PD_: Predevelopment runoff rate (m^3^/5 min)
RWH: Rainwater Harvesting
S: Tank storage capacity (m^3^)
SQ_50_: Median peak flow of a sample of significant events (l/s/ha)
SE_R50_: Median retention efficiency of a sample of significant events (−)
SE_CQ50_: Median inflow control efficiency of a sample of significant events (−)
T_CQ_: Annual time above predevelopment runoff (hours/year)
V: Volume of water in the tank (m^3^)
Y: Yield (m^3^/5 min)

## 1 Introduction

**Fig. 1:**
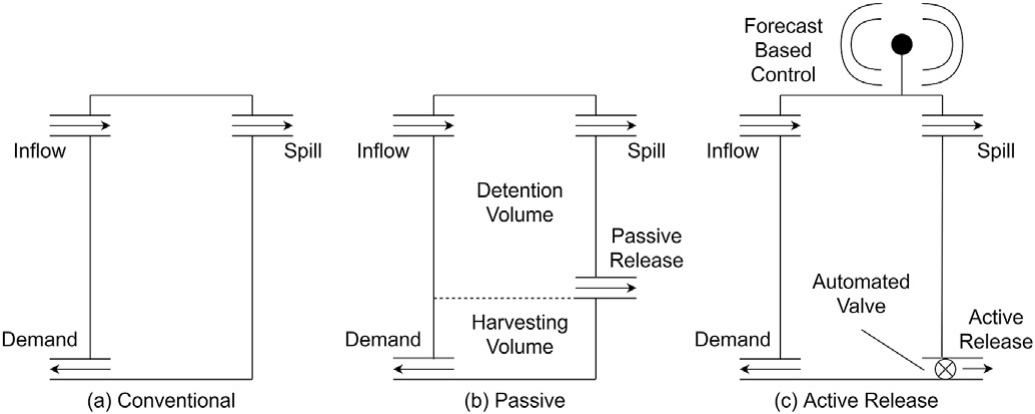
Configurations of three types of RWH systems. Adapted from Xu et al. (2018).

Previous research on domestic rainwater harvesting (RWH) has centred primarily on the ability of systems to deliver a reliable water supply (Abdulla and Al-Shareef, 2009 Helmreich and Horn, 2009; Roebuck et al., 2011). In recent years this focus has shifted to include stormwater management potential, which is often quantified as retention, the total captured volume over a given time interval (Burns et al., 2015; Campisano et al., 2017; Palla et al., 2017; Xu et al., 2018). Including stormwater management as a critical objective has led to a diversification of RWH system designs, with examples displayed in Fig. 1. Conventional RWH systems (Fig. 1a) are designed primarily to maximise water supply (The British Standards Institution, 2018). As such, they may be full at the onset of significant events, rendering them ineffective at reducing runoff. Alternative systems include an outlet to drain stored water which frees storage space in advance of rainfall events. Passive release systems (Fig. 1b) partition the tank into a water supply harvesting volume and a stormwater detention volume with a slow-release discharge outlet. Controlled release occurs when the water level is above the passive outlet (Fig. 1b) and the rate is determined entirely by water level and the size of the orifice (Xu et al., 2018). Active systems (Fig. 1c) are remotely controlled to balance water supply and stormwater management functions. They use rainfall forecasts to manage the release of water according to expected inflows and available retention volume in the tank (Xu et al., 2018).

Even though water supply and stormwater management objectives are increasingly considered jointly for RWH design, there has been minimal investigation into how these two traditionally conflicting water management objectives might trade-off. Jensen et al. (2010) concluded there were no trade-offs in a study limited to conventional tanks, where storage size was the only design variable. With the emergence of more sophisticated designs involving additional (passive or active) release systems, this conclusion needs to be revisited to assess the performance of alternative designs and understand the potential trade-offs between them. For this, performance metrics that characterise the dual objective of RWH tanks in a more complete and nuanced way are essential. While a wide range of metrics to quantify the stormwater management performance of RWH systems exist (Gerolin et al., 2010; Xu et al., 2018), they typically provide long-term averages. For example, Xu et al. (2018) evaluated both retention and water supply efficiency and frequency using an 11-year time-series, quantifying retention as the percentage of total roof runoff captured. Their findings indicated that active systems performed better with regards to baseflow restoration and stormwater retention, with minimal adverse impact on water supply, compared to the passive system. These results, however, were limited to a single stormwater management metric (retention) evaluated for the total simulation period. They did not consider performance during specific, extreme, storm events. Recent large-scale modelling efforts, such as the study of a sewer catchment in Palermo by Freni and Liuzzo (2019) and the catchment response framework developed by Jamali et al. (2020), also characterise the stormwater management of RWH systems using metrics averaged on an annual or longer time scale, similar to what is commonly done for water supply metrics.

Several field studies focusing on the dual use of RWH systems exist (e.g., DeBusk et al., 2013; Gee and Hunt, 2016; Braga et al., 2018). For RWH systems connected to high use commercial properties, these studies included evaluations of conventional (DeBusk et al., 2013), passive (Gee and Hunt, 2016) and active release (Gee and Hunt, 2016; Braga et al., 2018) systems. They quantified stormwater management performance based either on averaged per-event responses, e.g. average event overflow volume (Braga et al., 2018) or overall volume reduction (Gee and Hunt, 2016). However, these field studies found that the monitored retention provided by these systems varied dramatically between events, depending on antecedent storage and rainfall patterns.

These results stress the importance of understanding the retention of these systems during events with a return period of a year or more. These are the most likely to cause flooding, and to damage river morphology and ecosystems (Woods-Ballard et al., 2015). Event-based metrics are needed to capture the potential of RWH systems to bring about stormwater management benefits. A similar approach has been applied to other “green” stormwater management infrastructure such as green roofs (Gerolin et al., 2010; Woods-Ballard et al., 2015; Stovin et al., 2017). In extreme events, the flood mitigation potential of a stormwater management device depends on its ability to control both the total volume released and the peak rate of flow (Woods-Ballard et al., 2015). Gee and Hunt (2016) described the peak flow attenuation of both a passive and an active release RWH system during an observed event comparable to a 1-year 24-h design storm. However, Gerolin et al. (2010) highlighted the lack of robustness of this metric to antecedent storage conditions and the timing of peak rainfall intensity during a real-world storm. For example, if the peak intensity occurs late in an event; the tank may already be full and offer no peak reduction. Because of this, event-based metrics need to be based on a robust sample of storms rather than on a single extreme or design storm event. Both Stovin et al. (2017) and Gerolin et al. (2010) have argued that flow duration curves, corresponding to the system response to long continuous rainfall time-series, provide a more detailed evaluation of the system’s performance compared with single event detention metrics.

The absence of event-based stormwater management performance metrics for RWH has implications for engineering design guidance and practice. For instance, in the UK, design guidance on dual-use RWH system design is provided by the Sustainable Drainage System (SuDS) Manual (Woods-Ballard et al., 2015). This guidance is based on a previous engineering guideline, the British Standard (The British Standards Institution, 2013), which recommended adding enough storage volume to capture a 1:100-year event to a system designed to provide water supply. Such guidelines could lead to oversized tanks, but this conservative design approach may partly be due to the lack of performance metrics able to capture detention performance. Detention performance metrics need to refer to the system’s ability to limit discharge to a predevelopment rate, i.e., the flow rate before urbanisation.

To address this gap, and its implications for engineering practice, this paper develops a framework of metrics to adequately characterise the water supply and stormwater management performance of RWH systems. The paper achieves this using both multi-decadal time-series of continuous rainfall inputs and a storm event-based approach. It defines multiple performance metrics for RWH systems and uses them to undertake a multi-criteria visualisation of alternative designs. We also disaggregate the stormwater management metrics we propose on an event-by-event basis to explore the relationship between individual events and long-term performance in more depth.

## 2 Methodology

### 2.1 Case-study application

The purpose of this case study is not to determine which rainwater harvesting (RWH) system design is best, but to illustrate how nuanced stormwater management performance metrics may inform design decisions.

#### 2.1.1 System configurations

We consider the four system configurations used in Xu et al. (2018) because they cover the main categories of RWH system available: (1) **Conventional** system; (2) passive release system with 75% detention volume (**Passive 1**); (3) passive release system with 25% detention volume (**Passive 2**); (4) **Active** system. Two different **Passive** systems are chosen to examine a system where stormwater detention is prioritised (75% detention volume) and one which favours water supply (25% detention volume). To facilitate comparison between alternative designs, tanks modelled in this study all have a capacity of 1000 L, in line with the British Standard (The British Standards Institution, 2018) recommendations for RWH systems that provide water supply. The demand is assumed to be toilet flushing and clothes washing for an average British household of 2.4 people (Office for National Statistics, 2019); this results in daily usage of 120 l (The British Standards Institution, 2013). The roof area is 30 m^2^. The tanks are cylindrical with a diameter:height ratio of 4:3 for stability.

#### 2.1.2 Climatic data

We illustrate this approach using climatic inputs which were taken from the UK Climate Projections, as detailed in Stovin et al. (2017), (UKCP09, http://ukclimateprojections.defra.gov.uk/). The data is a 30-year data set incorporating climate change projections that has been disaggregated into 5-min time steps using STORMPAC (WRc, 2009). This time series is representative of a plausible mid-term future climate (2050) in Sheffield, UK.

Temporal resolution is an essential consideration for the quantification of peak outflow rates. Although an hourly timestep is appropriate for retention studies, it does not permit the modelling and interpretation of the detention performance of stormwater management devices (Stovin et al., 2017). In this case, utilising a dataset with a 5-min time step enables us to quantify both the retention and detention performance of these systems.

### 2.2 Modelling framework

#### 2.2.1 Conventional system model

A model was constructed to continuously simulate the behaviour of three types of household-scale RWH system (Fig. 1). We model each system using a Yield-After-Spillage (YAS) approach, which is the most conservative method of simulating RWH system behaviour (Fewkes and Butler, 2000). The model converts rainfall to stormwater runoff (tank inflow) based on a roof area of 30 m^2^ assuming an initial loss of 0.2 mm with a 2-h antecedent period and an additional 0.2 mm/day (Xu et al., 2018). For **Conventional** systems:

(1) $$Q_{\Delta t}=V_{t-1}+I_{\Delta t}-S$$

(2) $$Y_{\Delta t}\;=min\left\{ \begin{array}{l} D_{\Delta t}\\ V_{t-1} \end{array}\right.$$

(3) $$V_{t}=min\left\{ \begin{array}{l} V_{t-1}+I_{\Delta t}-Y_{\Delta t}\\ S-Y_{\Delta t} \end{array}\right.$$

where Q_Δt_ is the tank outflow, I_Δt_ is tank inflow, D_Δt_ is the demand, Y_Δt_ is the yield during the timestep Δt, V_t_ is the volume in-store at time t and S is the tank storage capacity.

#### 2.2.2 Passive system model

For the **Passive** systems, controlled release occurs before yield, resulting in a modified outflow (Q_Δt_) equation:

(4) $$Q_{\Delta t}=max\left\{ \begin{array}{l} 0\\ V_{t-1}+I_{\Delta t}+{\text{C}}_{\Delta t}-S \end{array}\right.$$

where C_Δt_ is the controlled release during the timestep Δt, which is calculated using the orifice equation:

(5) $$C_{\Delta t}=\mathrm{Cd}\left(\frac{1}{4}\pi d^{2}\right)\sqrt{2{\mathrm{gh}}_{t}}$$

where d is the equivalent outlet diameter, h_t_ is the head (m) acting over the centreline of the orifice at time t, Cd is the orifice discharge coefficient (Cd = 0.7 was adopted), and g is the acceleration due to gravity (9.81 m/s^2^). The passive release outlet is sized to deliver a maximum outflow of predevelopment runoff for a 1 in 30 year storm event equivalent to 5 l/s/ha, which results in a diameter of 0.0024 m (**Passive 1**) and 0.0032 m (**Passive 2**). We acknowledge that practical issues would prohibit such small diameters, and other forms of restriction would be necessary to achieve the low flow rate required. For example, a pressure-independent dripper could be used to achieve the required flow rate (Xu et al., 2018). This rate was calculated for the Sheffield area using HR Wallingford’s greenfield runoff rate estimation calculator, for this research a 1 in 30 year storm was specified (Kellagher, 2013). Yield is calculated using Eq. (2) and volume in the tank is calculated using Equation (6):

(6) $$V_{t}=min\left\{ \begin{array}{l} V_{t-1}+I_{\Delta t}-Y_{\Delta t}-{\text{C}}_{\Delta t}\\ S-Y_{\Delta t}-{\text{C}}_{\Delta t} \end{array}\right.$$

#### 2.2.3 Active system model

For the **Active** system, outflow, controlled release, yield and volume in the tank are calculated identically to the **Passive** system. There are many potential algorithms for determining emptying timing for the **Active** system; the method used by Xu et al. (2018) is implemented here. The controlled pre-storm release volume is the predicted overflow volume, which is determined by the difference between the available tank storage volume at the end of the previous day and predicted runoff volume for the following 24-h period. It is delivered through a 10 mm automated valve, driven by gravity (Xu et al., 2018). The model assumes a perfect rainfall forecast. The performance of active systems can be significantly affected by rainfall forecasting error. The main source of uncertainty is errors in rainfall intensity, which result in either over or under estimation of volume to be emptied (Xu et al., 2020). To simulate this potential inaccuracy, additional sensitivity analyses were undertaken in which a systematic bias of ± 10% was applied to the emptying volume for every event.

### 2.3 Performance metrics

As highlighted above, the stormwater management performance metrics adopted in previous studies often fail to capture all the information that may be relevant to the evaluation of these devices. Many have focused on long-term retention, rather than the event-based retention and peak runoff statistics that are most relevant for flood risk mitigation. Hence, we propose the following metrics to evaluate and compare each system’s overall performance comprehensively. The seven metrics chosen, and their equations, are presented in Table 1.

**Table 1:** Summary of performance metrics.

Metric	Symbol	Unit	Equation	Justification
**Long-term water supply**				

*Water supply efficiency*	E_WS_	-	${\text{E}}_{\text{ws}}=\frac{\sum {\text{Y}}_{\Delta \text{t}}}{\sum {\text{D}}_{\Delta \text{t}}}$ Range: 1 = good 0 = bad	Well-established volumetric water supply metric.

**Long-term stormwater management**				

*Retention efficiency*	E_R_	-	${\text{E}}_{\text{R}}=\left[1-\frac{\sum {\text{Q}}_{\Delta \text{t}}}{\sum {\text{I}}_{\Delta \text{t}}}\right]$ Range: 1 = good 0 = bad	Well-established volumetric stormwater retention metric.

*Inflow control efficiency*	E_CQ_	-	${\text{E}}_{CQ}=\left[1-\frac{\sum {\text{QC}}_{\Delta \text{t}}}{\sum {\text{I}}_{\Delta \text{t}}}\right]$ ${\text{QC}}_{\Delta \text{t}}=\left\{ \begin{array}{cc} {\text{Q}}_{\Delta \text{t},} & {\text{Q}}_{\Delta \text{t}}>{\text{Q}}_{PD}\\ 0, & otherwise \end{array}\right.$ Range: 1 = good 0 = bad	New metrics introduced here to quantify the system’s ability to control flow rates to a threshold that relates to the catchment’s predevelopment runoff characteristics.
			QC_Δt_ represents outflow above the predevelopment runoff rate, and Q_PD_ is the predevelopment runoff rate.	
*Annual time above predevelopment runoff*	T_CQ_	hour	${\text{T}}_{CQ}=\frac{\sum {\text{N}}_{\text{t}}}{12}$ ${\text{N}}_{\text{t}}=\left\{ \begin{array}{cc} 1, & {\text{Q}}_{\text{Δt}}>Q_{PD}\\ 0,\; & otherwise \end{array}\right.$	
			N_t_ is counted if the tank outflow is above the predevelopment runoff rate	
**Stormwater management during our sample of significant events**				

*Median peak outflow*	SQ_50_	l/s/ha	Median peak outflow over sample of significant events.	New metrics introduced here to quantify the system’s ability to control both flow rate and volume associated with high return-period events relevant for flood risk management
*Median retention efficiency*	SE_R50_	-	Median E_R_ over sample of significant events. Range: 1 = good 0 = bad	
*Median inflow control efficiency*	SE_CQ50_	-	Median E_CQ_ over sample of significant events. Range: 1 = good 0 = bad	

#### 2.3.1 Water supply

Two metrics have emerged as methods for determining the water supply performance of RWH systems: water supply efficiency and water supply frequency (Xu et al., 2018). *Water supply efficiency* (E_ws_) is a measure of the extent to which yield from the system meets volumetric demand.

Water supply frequency is a measure of the proportion of time when demand is met. Volumetric and time-based reliability are also common terms used to refer to water supply efficiency and frequency respectively (Mitchell et al., 2008). When there is a regular water demand, such as toilet flushing and clothes washing usage, both metrics are almost identical (<0.1% difference) (Xu et al., 2018). Therefore, to limit the number of metrics considered in this paper, we adopt the metric *Water supply efficiency* (E_ws_). If demand is highly variable in time, e.g. due to seasonal irrigation, drainage designers should also examine water supply frequency. It is acknowledged that the water supply will vary seasonally; for example, more water will be available in Winter as rainfall is greatest then. However, as the water available from these systems is supplementary to a constant piped supply, the overall E_ws_ enables an adequate comparison between the performance of different systems.

#### 2.3.2 Stormwater management

In terms of stormwater management, the most popular performance metric is overall *Retention efficiency* (E_R_). This metric quantifies water that is prevented from entering the drainage network.

This metric combines controlled releases (acceptable) and uncontrolled spills (potentially problematic). An alternative is to quantify outflow control using the predevelopment runoff rate calculated as the peak rate of runoff due to rainfall falling on a given area of vegetated land. In the UK this is defined as greenfield runoff and computed using a specific formula (Kellagher, 2013). We propose the metric, *Inflow control efficiency* (E_CQ_), which is defined as the proportion of inflow controlled to predevelopment runoff rate, to quantify this behaviour. The *Annual time above predevelopment runoff* (T_CQ_) in hours per year is also an important characteristic.

The ability of these systems to control outflow rates must also be measured on a storm event basis. Previous quantifications of stormwater detention by RWH systems have been limited to peak flow attenuation for specific events, which, as discussed in the introduction, is not a robust metric. Instead, we propose basing this metric on a sample of relevant events, specifically the set of ‘significant’ events. In what follows, the sample size of this set was selected based on the return period of interest and on time series length. Here, we selected the 30 most significant events over a 30-year time-series to have an empirical sample of events that are indicative of the 1:1-year event. Events with an annual return period are of interest to drainage engineers as they can cause morphological damage to the catchment (Woods-Ballard et al., 2015). Although SuDS can reduce the frequency and/or severity of flooding, their impact on large events may be minimal. As such we chose not to develop specific metrics for events with return periods higher than one year. What defines a ‘significant’ event depends both on catchment characteristics and on drainage guidance and regulations. We considered events with the largest 1-h, 6-h and 24-h rainfall depth as alternative definitions for our ‘significant’ events. The characteristics of these events are contained in the Supplementary Data.

For each of these 30 significant events, we determined the peak 5-min outflow rate and determined the *Median peak outflow* (SQ_50_). To address the requirement to quantify volumetric control during these extreme events, the retention and proportion of inflow controlled to predevelopment runoff rate create two further metrics: *Median retention efficiency* (SE_R50_) and *Median inflow control efficiency* (SE_CQ50_).

### 2.4 Multi-criteria visualisation

Our multi-criteria visualisations aim to examine the potential trade-offs between the metrics that reflect different aspects of RWH systems’ use for water supply and stormwater management. For this, we need to compute and represent all the metrics identified in Section 2.3 for each of the four RWH systems. We use two visualisation techniques to convey this information: a parallel plot and a radar plot. Both are fit for representing multiple metrics concurrently by attributing one axis to each metric, and having axes represented either in a parallel way (parallel plot) or radially (radar plot). In general, all axes in a plot use a common convention to rank alternatives from worst performing to best performing. For instance, performance will increase from bottom to top in our parallel plot and from the outside towards the centre in our radar plot. In this work we demonstrate both visualisations with slightly different specifications, but it is important to remember that both can be used interchangeably in practice, with parallel plots being particularly suited for cases where there is a large number of alternatives (e.g., Woodruff et al., 2013). When a design alternative A is equal to or better than B with respect to all metrics, there is no tradeoff to consider, and we say that A dominates B in the Pareto sense. Otherwise, the visualizations provide drainage engineers and stakeholders alike with a transparent and at-a-glance way to determine trade-offs between alternatives (e.g., Kasprzyk et al., 2016). A possible next step once equipped with these metrics is to aggregate them through weighted sum as part of a multiattribute decision making process (Clemen and Reilly, 2013). However, this work aims at providing a template for extracting and visualising the information for dual-use RWH design decisions, rather than prescribing how these metrics should be used to reach a design decision. Besides, there exist well-documented, severe challenges to aggregating metrics in an unbiased way (Brill et al., 1990; Franssen, 2005; Woodruff et al., 2013), especially in the type of multi-alternative, multi-stakeholder context that corresponds to choosing and implementing RWH systems in a flood- or drought-prone community.

### 2.5 Sensitivity analysis

The demand fraction is a dimensionless ratio given by annual demand divided by annual runoff (Fewkes and Butler, 2000). For the case presented in Section 2.1, the mean annual runoff for the 30-year time series was 683.1 mm, such that the modelled demand fraction was 2.14. A sensitivity analysis was performed to examine the impact of different demands on the performance metrics discussed in Section 2.3. Here we maintain a constant roof area and rainfall and vary the household water demand to generate a range of demand fractions from 0 to 5.0.

A similar approach was used to examine the sensitivity of performance to storage volume. Here, the dimensionless ratio, storage fraction (given by the storage volume divided by annual runoff, Fewkes and Butler, 2000) was varied between 0 and 0.20 to simulate a range of tank sizes between 0 and 4.1 m^3^.

### 2.6 Long-term and significant event-based stormwater management performance

#### 2.6.1 Flow duration curve

**Fig. 2:**
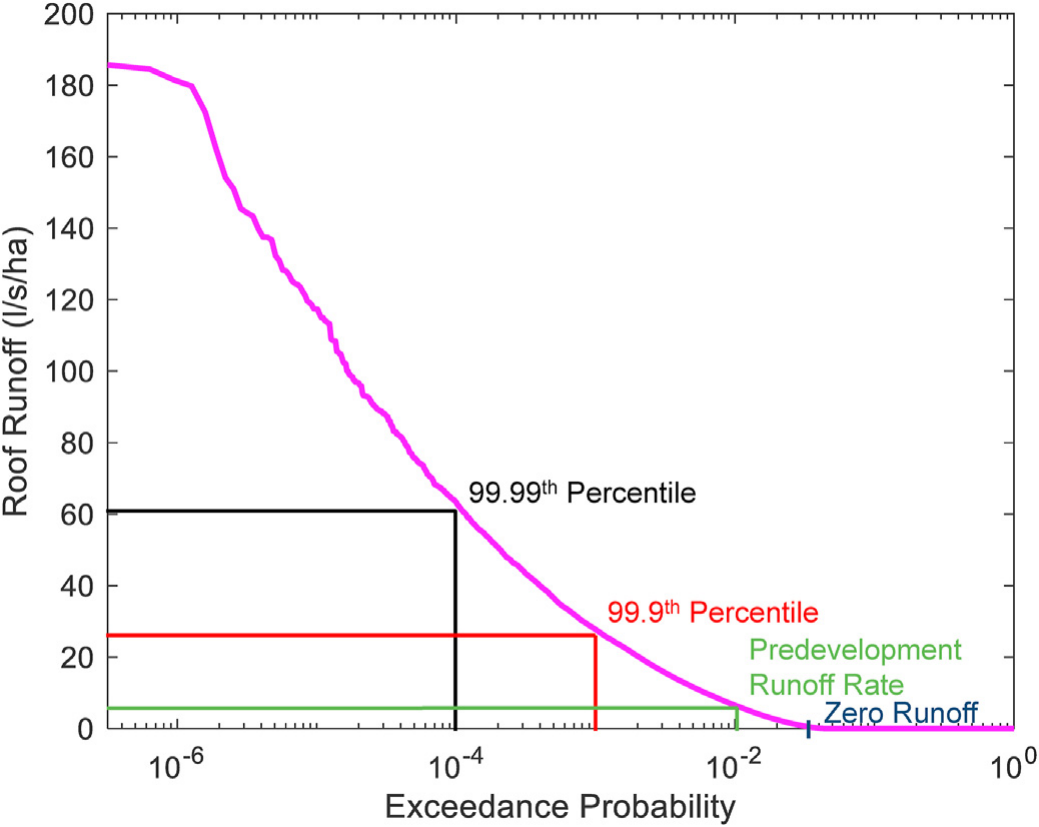
Example of flow duration curve for the 30-year time series.

As the peak outflow is determined on a 5-min basis, it can be very sensitive to local fluctuations in the rainfall rate. Therefore, we complement our analysis with a graphical approach in the form of the flow duration curve (Stovin et al., 2017). A flow duration curve is a plot of runoff vs the proportion of time that a runoff is equalled or exceeded. It is calculated by determining the exceedance probability of each of the tank outflow rates. Fig. 2 shows an example of a flow duration curve for the long term 30-year roof runoff. The largest roof runoff observed during the 30-year time series is 185 l/s/ha, which is equivalent to 67.2 mm/h.

A flow duration curve is typically used to show performance over the total simulation period or individual events. It displays a range of information useful to drainage engineers and facilitates comparisons between different systems.

#### 2.6.2 Significant event-based performance

In addition to the median significant event performance metrics, consideration of the distribution of performance outcomes across the full set of 30 significant events may lead to additional insights. In this section, we examine the peak outflow, E_R_ and E_CQ_ for the largest 30 events with a 1 year return period, as determined in Section 2.3.

## 3 Results

### 3.1 Performance metrics and multi-criteria visualisations

Table 2 shows the water balance per m^2^ of roof area and the performance metrics for each system. The initial losses from the roof are 19% of the rainfall, in line with Mentens et al. (2006) who reported 19% retention of rainfall by non-greened roofs in Brussels. The water demand is over double the volume of roof runoff, which indicates that, regardless of the rainwater harvesting (RWH) system size, the maximum *Water supply efficiency* (E_WS_) is limited to 0.46. This value is only marginally larger than the best performing systems (**Active** and **Conventional**) at 0.42. Outflow and Outflow above predevelopment runoff are almost identical for both the **Active** and **Conventional** systems. For both **Passive** systems, the Outflow above predevelopment runoff rate is significantly lower than total Outflow, illustrating their capacity to limit the high outflow rates associated with uncontrolled spills. The **Passive 2** (25% detention volume) system seems to perform better of the two, as it has less Outflow but comparable Outflow above predevelopment runoff rate to the **Passive 1** (75% detention volume) system.

**Table 2:** Annual average water balance and performance metrics.

Water Balance						
		No System	Conv	Passive 1	Passive 2	Active
Rainfall (m^3^/year/m^2^)		0.84				
Initial losses (m^3^/year/m^2^)		0.16				
Roof runoff (m^3^/year/m^2^)		0.68				
Demand (m^3^/year/m^2^)		1.46				
Yield (m^3^/year/m^2^)		0	0.62	0.46	0.59	0.61
Outflow (m^3^/year/m^2^)		0.68	0.06	0.22	0.09	0.07
Outflow above predevelopment runoff rate (m^3^/year/m^2^)		0.53	0.06	0.02	0.02	0.07

**Performance Metrics**						

**Long-term water supply**						

Water supply efficiency (E_ws_) (−)		0	0.42	0.32	0.40	0.42

**Long-term stormwater management**						

Retention efficiency (E_R_) (−)		0	0.91	0.68	0.87	0.90
Inflow control efficiency (E_CQ_) (−)		0.22	0.91	0.97	0.97	0.90
Annual Time above predevelopment runoff (T_CQ_) (hours per year)		109.39	8.65	1.25	2.25	4.28

**Stormwater management during our sample of significant events**						

Median peak outflow (SQ_50_) (l/s/ha)	1-h	83.5	19.1	4.6	4.6	23.5
	6-h	89.2	61.7	28.5	47.4	63.9
	24-h	72.2	64.8	40.5	50.9	87.5
Median retention efficiency (SE_R50_) (−)	1-h	0	0.85	0.60	0.80	0.87
	6-h	0	0.59	0.48	0.55	0.65
	24-h	0	0.49	0.47	0.49	0.58
Median inflow control efficiency (SQ_CQ50_) (−)	1-h	0.03	0.87	1	1	0.89
	6-h	0.03	0.61	0.90	0.77	0.66
	24-h	0.05	0.50	0.87	0.74	0.59

In terms of the significant events, for each of the metrics the order of performance from best to worst remains constant regardless of the time period over which the largest rainfall depth was calculated. The **Passive** systems are better at reducing peak flow and limiting flow to the predevelopment rate whereas the **Active** and **Conventional** system have larger retention values. For all performance metrics, all systems perform best during the events with the worst 1-h volume as these generally have lower durations and total volumes. The SuDS Manual (Woods Ballard et al., 2015) identifies events with the largest volume during a 6-h period as of critical importance; the metrics for these events will be presented throughout the rest of this paper with the metrics for the 1-h and 24-h largest volume events available in the Supplementary Data.

The systematic bias applied to the emptying volume resulted in a negligible impact on the performance metrics, with less than a 3% difference between the cases for all metrics. This is due to the size of the storage volume; as the large events usually necessitate large emptying volumes, a 10% variation will not make a significant difference. In addition, as emptying is required infrequently, the impact on the average water supply and retention efficiencies is minimal.

**Fig. 3:**
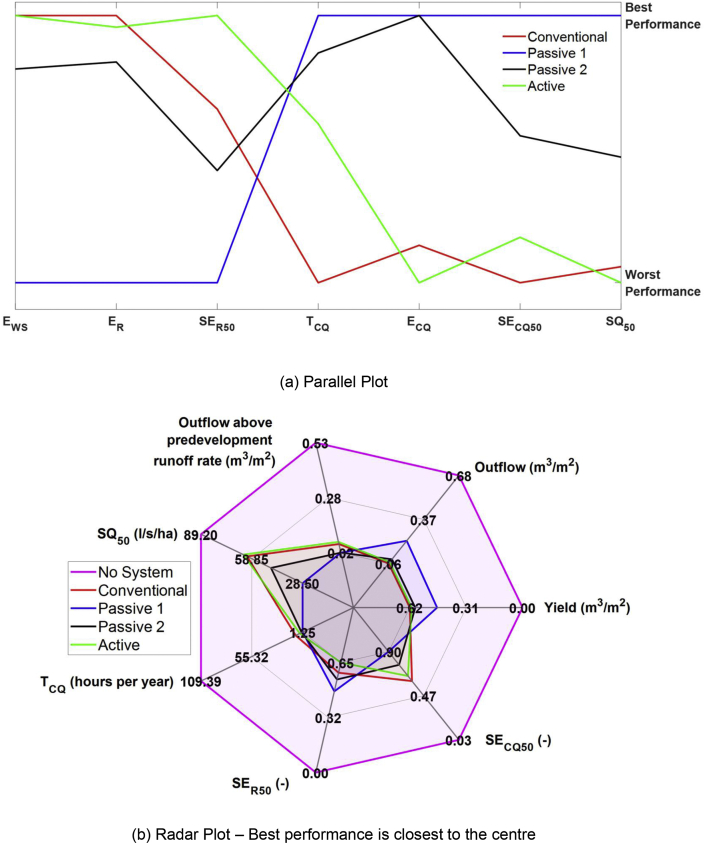
Multi-criteria Visualisations.

Fig. 3 presents a parallel plot and a radar plot intended to convey the conflicting rainwater harvesting objectives of water supply and stormwater management. The parallel plot (Fig. 3a) shows all values with an axis normalised and constrained to the best and worst performance. From both plots, no system exhibits Pareto dominance over the others. If the objectives are E_WS_ and *Retention efficiency* (E_R_), both **Conventional** and **Active** systems are best. If the *Inflow control efficiency* (E_CQ_) and *Median peak outflow* (SQ_50_) are of concern, the **Passive 1** system reduces the largest quantity of flow to below predevelopment runoff. What is more, all variables lead to a different ranking of alternatives; this illustrates the metrics we propose provide complementary insights into system performance. The radar plot (Fig. 3b) shows similar information to the parallel plot but using absolute values for the outflows, yield and T_CQ_. The priorities of the drainage designer will vary, so it is impossible to recommend one system type universally. The **Passive 1** system’s control of outflow rates is again highlighted, both overall and during extreme events.

### 3.2 The impact of the demand fraction on system performance

**Fig. 4:**
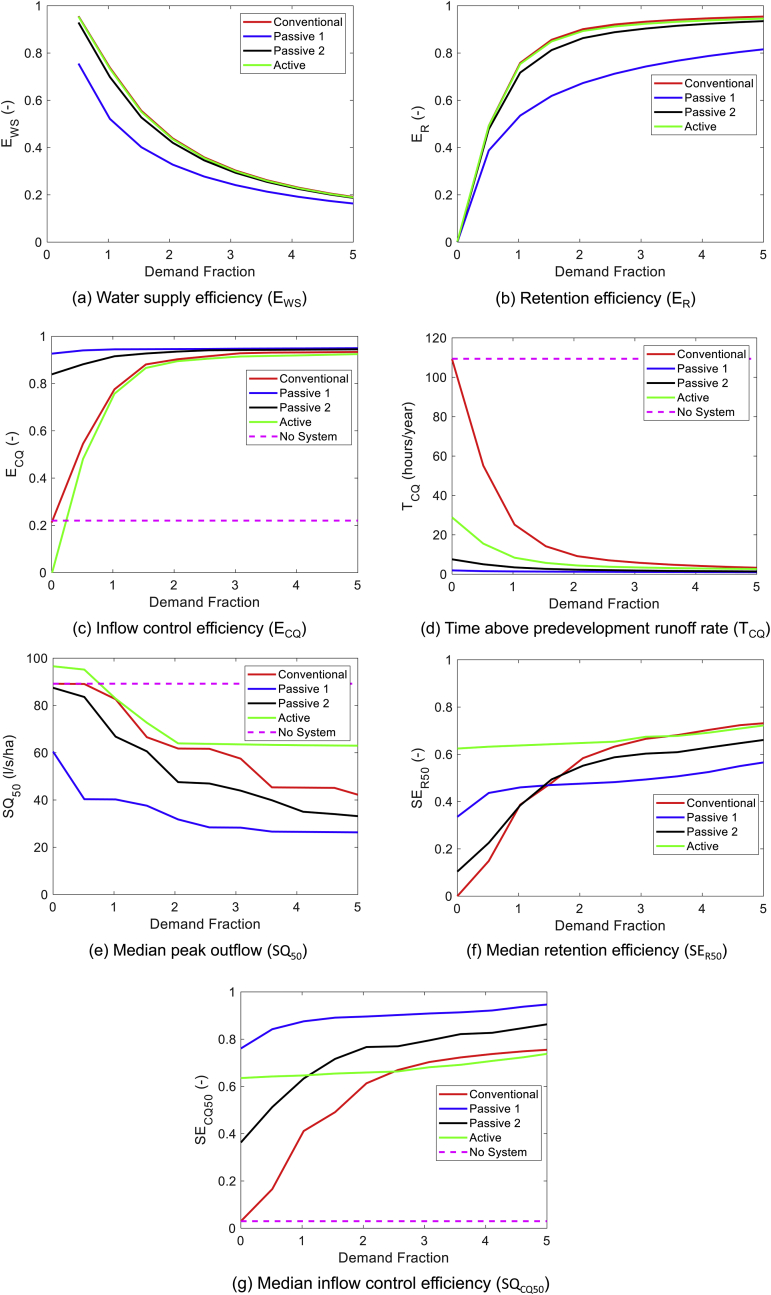
Demand Sensitivity Analysis.

A sensitivity analysis is used to examine the impact of demand variation on performance. Conventionally used metrics such as E_WS_ (Fig. 4a) and E_R_ (Fig. 4b) are the most sensitive to demand for all systems. For the E_CQ_ (Fig. 4c), both **Active** and **Conventional systems** vary more with demand than the **Passive** systems. For the SQ_50_ (Fig. 4e), the peak flow decreases consistently with demand for the **Conventional** and **Passive 2** systems. For the **Passive 1** and **Active** systems, there is a large decrease in peak outflow at a demand fraction of approximately 1.5; the outflow rate remains almost constant after this. For all demand fractions examined, the **Passive 1** system has the lowest SQ_50_.

For both the SE_R50_ and the E_CQ_, the **Active** and **Passive 1** systems perform relatively consistently across the demands. In contrast, the performance of both the **Conventional** and **Passive 2** systems increase steadily with increasing demand.

The comparative performance of the different systems is relatively insensitive to demand fraction, with the **Passive 1** system performing consistently well for all runoff rate metrics in the range 0.0 < demand fraction < 5.0.

The sensitivity analysis for the storage fraction is presented in the Supplementary Data. For all systems and metrics, the performance improved as the storage increased. The difference in performance between systems also decreased with increasing storage volume.

### 3.3 Long-term and significant event-based stormwater management performance

#### 3.3.1 Flow duration curve

**Fig. 5:**
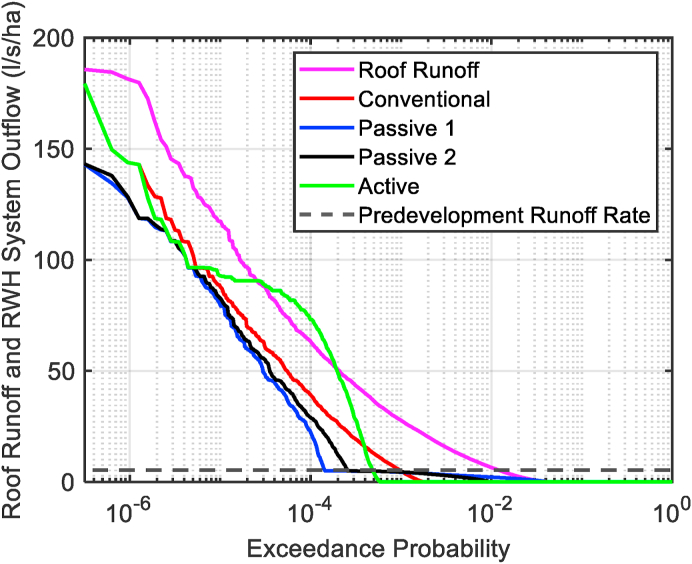
Flow duration curve.

The SQ_50_ discussed in the multi-criteria visualisation indicates stormwater detention performance. Yet it is still a single metric. A more comprehensive evaluation of stormwater detention performance is the flow duration curve which allows a comparison to be made across all systems and storm events (Fig. 5).

Fig. 5 shows that roof runoff occurs for 4% of the simulation time. Similarly, without any intervention, the roof runoff would exceed predevelopment runoff rates approximately 0.8% of the simulation time. Higher runoff rates are exceeded for less time. From the flow duration curve, the **Active** system has the lowest time above zero discharge. However, the emptying of these systems causes a controlled outflow greater than roof runoff for 0.01% of the simulation time. It is crucial that the timing of this emptying occurs independently of storm events to ensure that the burden on drainage systems is not increased. The **Passive 1** system has the lowest T_CQ_, only exceeding this threshold for 0.015% of the time. However, the **Passive 1** system also has the highest proportion of time above zero discharge, longer even than the roof runoff. The **Passive** systems perform best at peak runoff reduction; **Active** and **Conventional** systems perform comparably. It is clear from this demonstration that the flow duration curve successfully complements other metrics in describing the year-round behaviour of these systems.

#### 3.3.2 Significant event-based performance

**Fig. 6:**
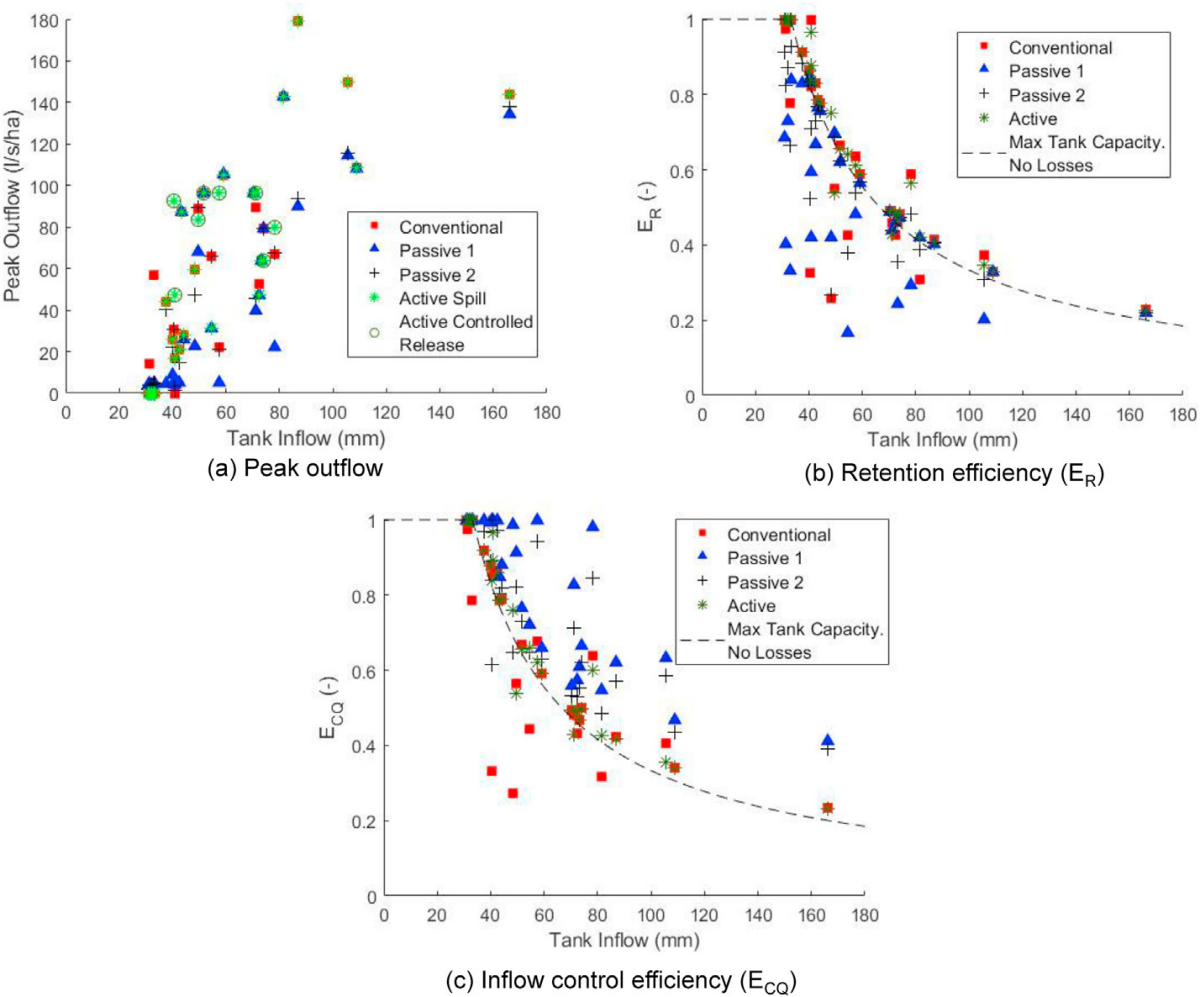
Scatterplots of system performance during significant events.

Fig. 6 shows the complete set of peak outflows, E_R_ and the E_CQ_ for the 30 most ‘significant’ storm events in the 30-year time series. One thing that is very clear from these plots is the significant spread of individual event metrics around the median values reported in Table 2. The degree of scattering reflects the influence that antecedent conditions and individual storm event characteristics have on performance during a specific event. By definition, median metrics do not represent the true variability of expected performance. For example, Fig. 6b highlights the fact that, while the SE_R50_ for the **Conventional** system is 0.59, its performance in individual significant events could be anything between 0.25 and 1.0.

Peak outflow is shown in Fig. 6a. As **Active** systems can have large controlled releases as discussed in Section 3.3.1, dedicated circles have been added on Fig. 6a to indicate occasions which are due to spill and which are due to active release. The figure shows that for smaller events, the **Passive 1** system has the lowest peak outflow. However, similarly to the E_R_ (Fig. 6b), as the events become larger, there is little distinction between the performance of the different systems.

For E_R_, Fig. 6b shows that, although all systems exhibit high E_R_ (>0.5) for the majority of events, there are a small minority which have E_R_ closer to zero. This variability again is due to antecedent conditions. For smaller events, significant disparities can be observed between the performances of the different systems, though there are smaller variations in performance between the system types for inflow events above 65 mm. The dashed line indicates the E_R_ associated with the maximum capacity of the tank (when empty) assuming no losses (i.e. no water supply during the event). Many points for both the **Conventional** and **Active** systems are on or above this line, as the household water demand during the event results in extra capacity for storage.

For the E_CQ_, Fig. 6c shows that in all events, the **Passive 1** system has the best performance. Again, the dashed line represents the maximum capacity of the tank with no losses. Most of the system performances fall above this line, illustrating the ability of these systems to control outflow rates, especially the **Passive 1** system; it is above this line for all events.

## 4 Discussion

This paper set out to provide a set of metrics which capture both the water supply and stormwater management performance of RWH systems. Some of these metrics (*Water supply efficiency* (E_WS_) and *Retention efficiency* (E_R_)) have been well established by previous literature. The existing metrics (E_WS_ and E_R_) do not provide an adequate representation of the stormwater management potential of these systems. For example, in the illustrative case study presented in Section 3.1, the **Conventional** system has both the highest E_R_ and E_WS_. However, it does not provide any additional control beyond its retention capacity (as indicated by the minimal difference between the E_R_ and *Inflow control efficiency* (E_CQ_) metrics). To quantify the release control capacity of these systems, we examine their ability to reduce inflow to the predevelopment runoff rate via the metrics: E_CQ_ and *Annual time above predevelopment runoff* (T_CQ_). The selection of stormwater management performance metrics reflects the two most common needs of receiving drainage systems: either complete retention or flow control. In cases where downpipe disconnection has occurred and the flow is either directed into waterways or sustainable drainage systems, metrics related to predevelopment runoff would be preferred as the high flow rate due to the active release may cause morphological damage. Conversely, where the RWH systems are connected to a combined sewer system, the focus would be maximising retention during extreme events and an **Active** system which empties reliably in advance of events would be preferable.

Each of the above metrics is presented as a long-term average over the 30-year time-series. It should be noted that this may hide seasonal variability for *E*_WS_, as lower values are expected during Summer months. In the UK, where RWH systems typically operate as an addition to mains water supply, an average quantification is an adequate method of determining water supply capability. Although average values are appropriate for water supply assessment, this is not the case for stormwater management, as performance during extreme events is of critical importance to the application of these systems as sustainable drainage devices. For example, the **Conventional** system has an overall *E*_R_ of 0.91, which is significantly higher than the *Median retention efficiency* (SE_R50_) of our sample of ‘significant’ events (0.59). Using the overall E_R_ metric alone may lead to an overestimation of stormwater management performance during extreme events. This finding led to our identification of three further metrics based on our sample of 30 ‘significant’ events: *Median peak outflow* (SQ_50_), SE_R50_ and *Median inflow control efficiency* (SE_CQ__50_). These metrics show that the **Passive 1** system has the lowest SE_R50_ (0.48), (recall that **Passive 1** has a 25% percent retention capacity and 75% detention volume, whereas **Passive 2** has 75% retention capacity and 25% detention volume) and if in this instance performance during large events was of concern to drainage designers an **Active** system (SE_R50_ of 0.65) would be optimal. Such an assessment would not have been possible without the inclusion of these metrics.

We found that although the peak outflow provides a useful metric for assessment of the detention capability of different RWH systems, it is very sensitive to antecedent conditions and the shape of individual storms. Therefore, the flow duration curve is proposed as a method to characterise the ability of these systems to limit stormwater runoff rate. There are many ways that this graph can be read, including the runoff rates that are exceeded for particular return periods (e.g. 99.99^th^, 99.9^th^ percentile) or the duration of time for which a specified runoff rate (e.g. zero or predevelopment runoff) is surpassed. Probabilistic approaches are used to set regulatory requirements for river water quality, with set 90 and 99^th^ percentile thresholds for biological oxygen demand levels (The Foundation for Water Research, 2019). However, for stormwater runoff, these thresholds would be lower due to the intermittent nature of rainfall. The decision as to what the key thresholds should be is expected to be dependent on the receiving catchment’s hydrological response. One possible option we explored is the T_CQ_. This value is of concern to drainage engineers, as current guidance places a heavy emphasis on limiting runoff above this value (Woods-Ballard et al., 2015). In addition to determining exceedance threshold values, the flow duration curve can be used to identify the impact of different active emptying algorithms on runoff rates. In this study, high rates of outflow from the **Active** system, which exceed roof runoff, are observed for 0.01% of the simulation time. This proportion of time only equates to 1 h per year for this system but could potentially be longer for smaller tanks which would empty more often.

The purpose of the case study is not to determine the best system, but to illustrate the ability of the proposed framework to capture all nuances of dual-function RWH system behaviour. Our multi-criteria visualisation highlights the importance of the procedure used to determine the emptying of the **Active** system (in this case, every 24 h as needed). It can empty during rainfall events, causing an increase in peak outflow and a decrease in retention. Note that this algorithm (proposed in Xu et al., 2018) assumes perfect day-ahead rainfall forecasts. Real-world **Active** systems are even more challenging to implement using imperfect forecasts. This can result in either a tank that is too full (resulting in spills) or too empty (resulting in subsequent supply shortage) before a storm. Although the systematic bias applied to the **Active** system’s emptying volume did not significantly alter results, further strategies to improve the capacity of the **Active** system to mitigate flood risks and control flow rate could be employed, such as reducing the active release flowrate and utilising 7-day rainfall forecasts (Xu et al., 2020) or adopting a minimum emptying time of 48 h before a storm (Woods-Ballard et al., 2015). Such strategies are also impacted by issues of forecast accuracy. Both the timing of these events and the availability of adequate forecasts are crucial, as one full active emptying of the tank would result in the equivalent of a 33 mm rainfall event over the space of 110 min. The methods presented in this paper could be used to examine the performance of different emptying strategies.

Currently, except for the analysis presented in Fig. 6, we draw no distinction between outflow attributed to spill or controlled release (passive or active), whereas Xu et al. (2018) calculated retention efficiency based on spill alone. This approach results in higher values of E_R_ for the **Passive 1**, **Passive 2** and **Active** systems than observed in our study. The impact that controlled releases from **Active** and **Passive** systems might have on the performance of the receiving drainage systems is unclear, so by separately identifying spill and controlled release, a fully informed assessment can be made. There is potential for discharging the controlled release to sustainable drainage systems, such as a swale or rain garden, as was done for the **Active** system examined by Gee and Hunt (2016). In many locations, this type of approach may not be possible due to space limitations or underlying soil conditions or due to high flow rates caused by the active release which may damage the morphology of the receiving water body.

A sensitivity analysis concerning household water demand is essential as it is often assumed that householders will exclusively use rainwater for their non-potable water needs when available. However, Quinn et al. (2020) showed that householders with a downstairs toilet connected to a conventional rainwater harvesting system did not use the water available to them as often as would be expected by the British Standards Institution (2013). In the case of the **Passive 1** and **Passive 2** systems, the E_CQ_ is still high for low demands. However, this is not the case for **Active** and **Conventional** systems. It is recommended that careful consideration is taken of demand during the design phase to ensure that it is accurate, and its variations considered when determining stormwater management impact.

The metrics, long-term performance assessment and sensitivity analysis presented in this paper are intended for use by drainage designers. Current RWH stormwater management guidance for the UK considers storage volume as the only design variable with no alternatives to conventional RWH systems designed exclusively for water supply. This approach leads to disproportionately large systems, e.g. 3 m^3^ for 30 m^2^ roof space, which makes RWH an unattractive option owing to space concerns. This paper has illustrated the effectiveness of a 1 m^3^ system at providing both water supply and stormwater management. Although no one system exhibited Pareto dominance, drainage engineers can utilize the multi-criteria visualisation to make informed drainage decisions that will reflect the preferences of home owners and local communities.

## 5 Conclusions

The potential for rainwater harvesting systems to provide both water supply and stormwater management is increasingly recognised, fostering interest in real-world applications and prompting a search for alternative designs. This paper supports these efforts by proposing the first set of metrics to fully quantify the stormwater management performance of RWH systems. Classic retention metrics have two key drawbacks: firstly they tend to focus on long-term volumetric performance rather than performance within specific, extreme, events; and secondly in treating uncontrolled spill and controlled outflow in the same way. We propose two metrics that measure a system’s capacity to control outflow below a threshold (e.g., runoff before urban development) by quantifying annual average volumes and times above this threshold. We also propose three robust metrics representing system response to severe rainfall events, by extracting a set of such events from a long-term time series, and taking the median across events of peak flow, retention efficiency, and outflow control efficiency. We combine these six metrics with a widely used measure of water supply efficiency to obtain a set of seven metrics, five of which are novel for RWH systems. Comparison of four alternative RWH system designs with these seven metrics computed over a 5-min resolution, thirty-year time series demonstrate that they provide complementary insights into overall design performance. Indeed, we use multi-criteria visualisation as a transparent and at-a-glance way to show that all metrics evaluate and rank alternatives differently from the others. We have also highlighted the value of a flow duration curve for capturing the system’s cumulative long-term performance. We suggest that a threshold such as time above predevelopment rate could be further developed as a regulatory requirement. This framework provides drainage designers with an easily applicable method for determining the benefits of individual rainwater harvesting systems to their catchment.

## Declaration of competing interest

The authors declare that they have no known competing financial interests or personal relationships that could have appeared to influence the work reported in this paper.
