# Supplementary material for: Quantifying the performance of dual-use rainwater harvesting systems
Source: Water Res X. 2020 Dec 13;10:100081. doi: 10.1016/j.wroa.2020.100081 (PMC7806874; doi:10.1016/j.wroa.2020.100081)
Supplement: Multimedia component 1 [file mmc1.docx]

**Supplementary Material**

Characteristics of Significant Events

| Event  Classification | Volume (mm) | | | | Length (hours) | | | |
| --- | --- | --- | --- | --- | --- | --- | --- | --- |
|  | Min. | Max. | Median | Mean | Min. | Max. | Median | Mean |
| 1 hour | 13.2 | 169.5 | 41.7 | 49.5 | 2.9 | 52.9 | 12.8 | 16.8 |
| 6 hour | 32.8 | 169.5 | 51.9 | 62.2 | 4 | 91.6 | 14.8 | 20.2 |
| 24 hour | 44.8 | 169.5 | 63.9 | 71.7 | 5.8 | 91.6 | 23.5 | 27.1 |


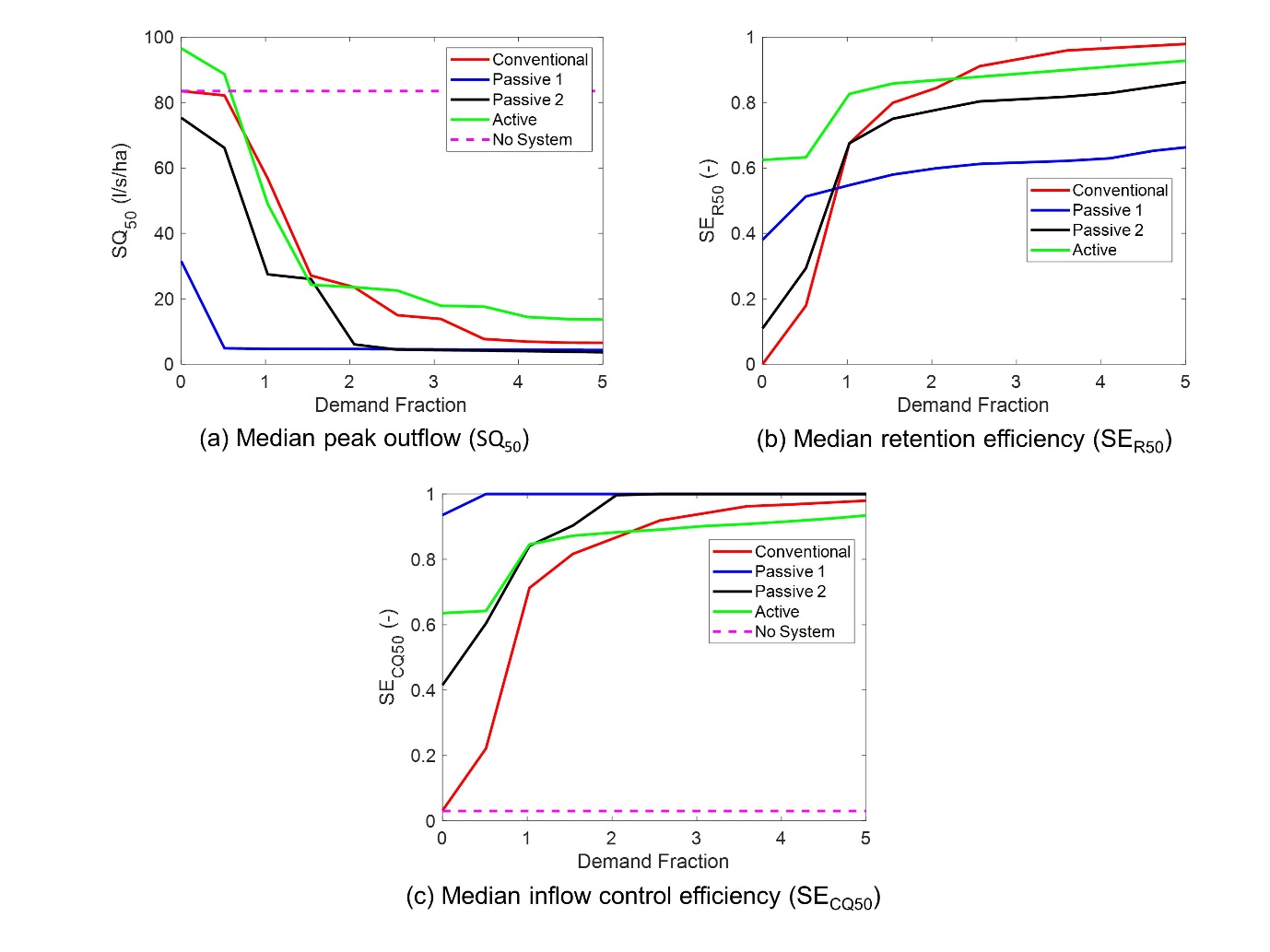


Demand Sensitivity Analysis for Events with the Largest 1h Rainfall Depth


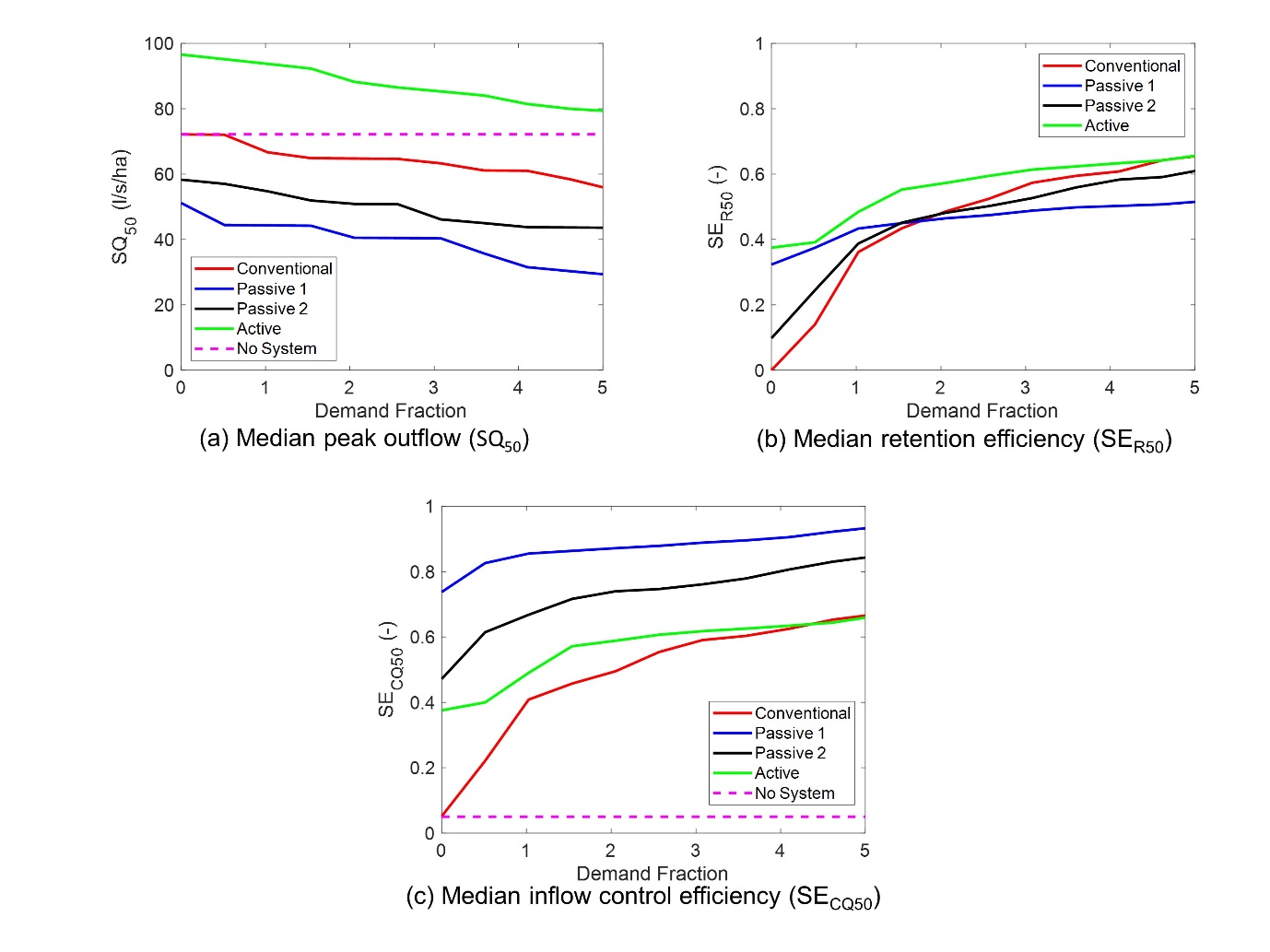


Demand Sensitivity Analysis for Events with the Largest 24h Rainfall Depth


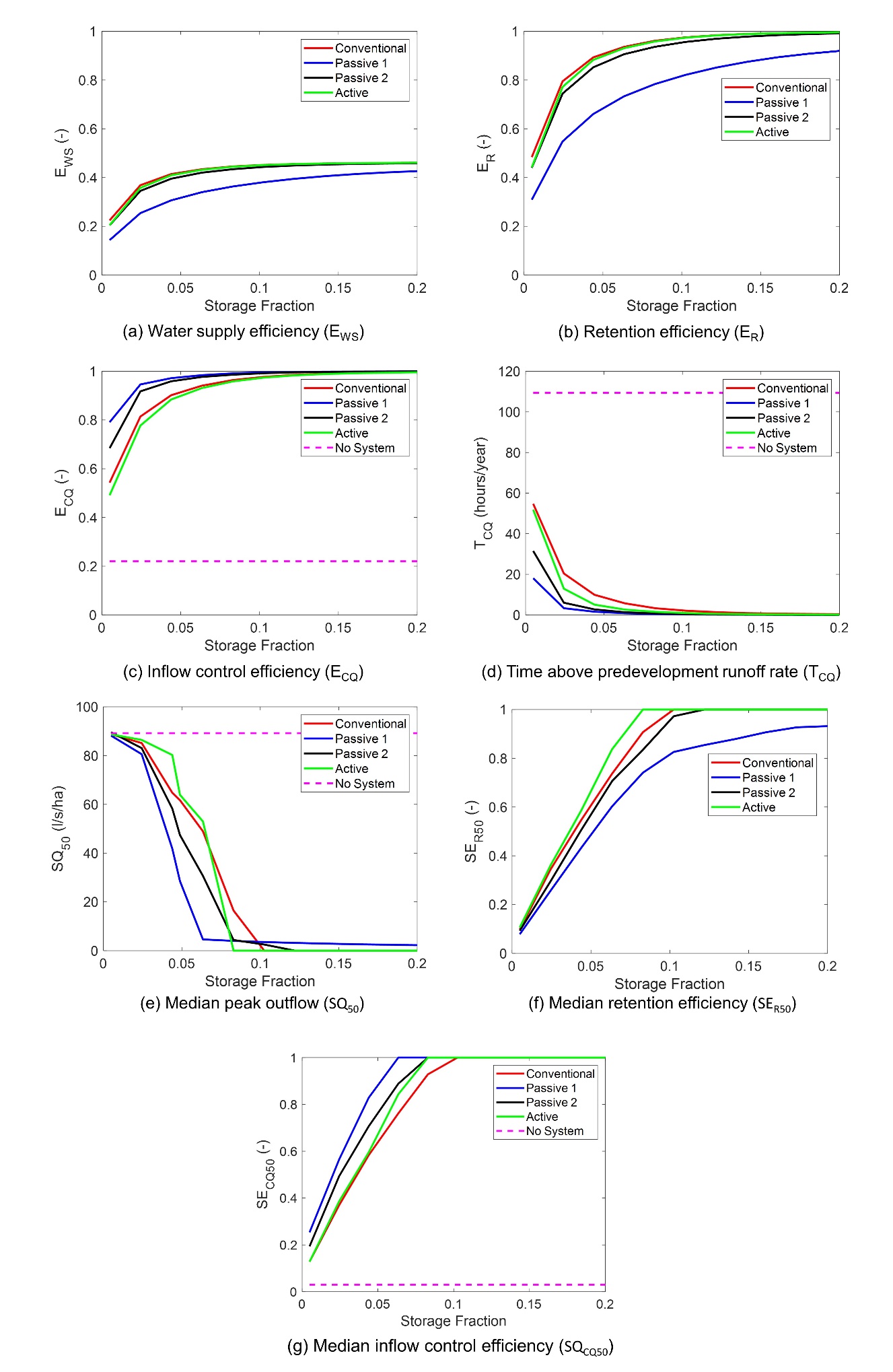


Storage Sensitivity Analysis


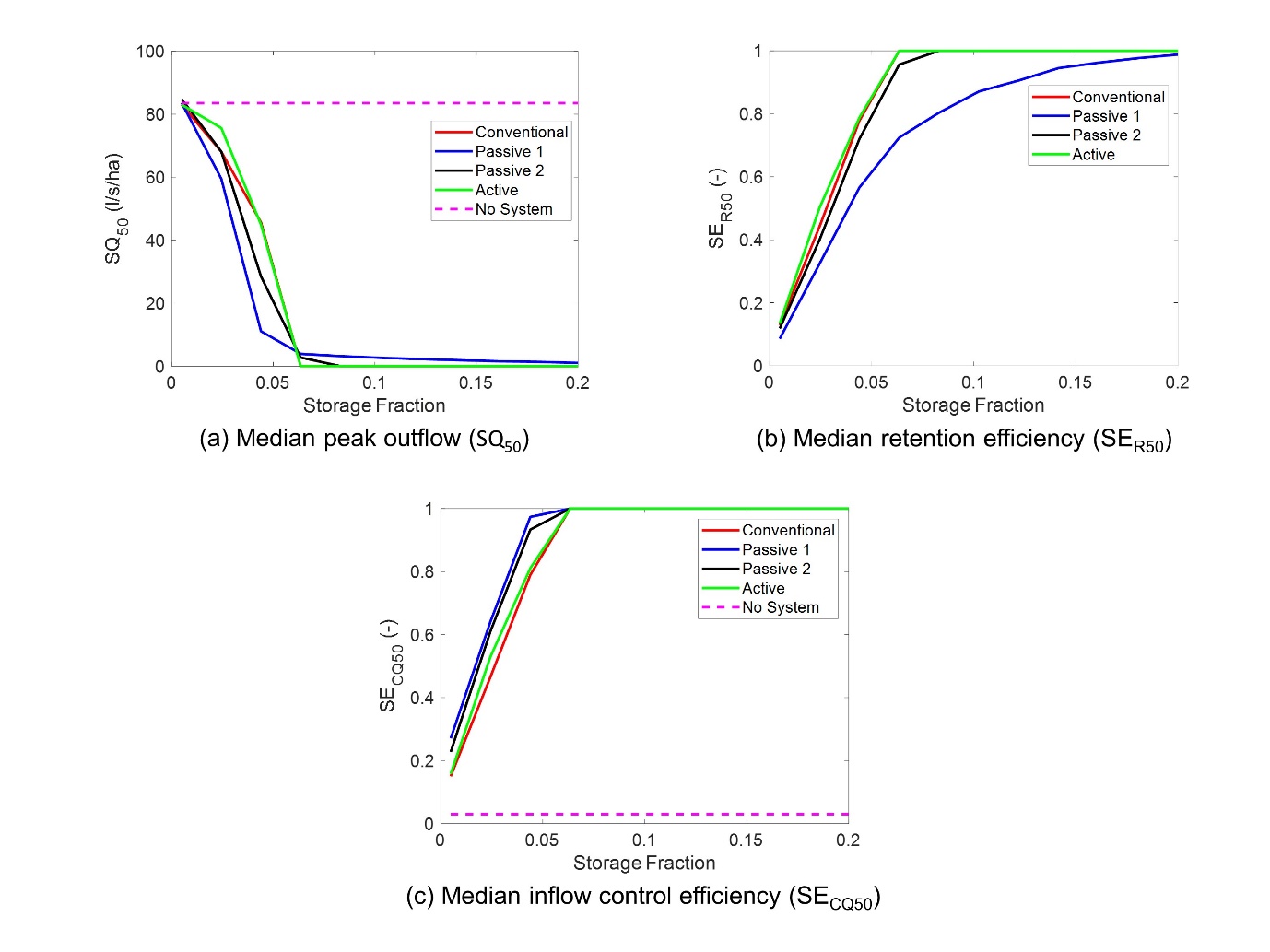


Storage Sensitivity Analysis for Events with the Largest 1h Rainfall Depth


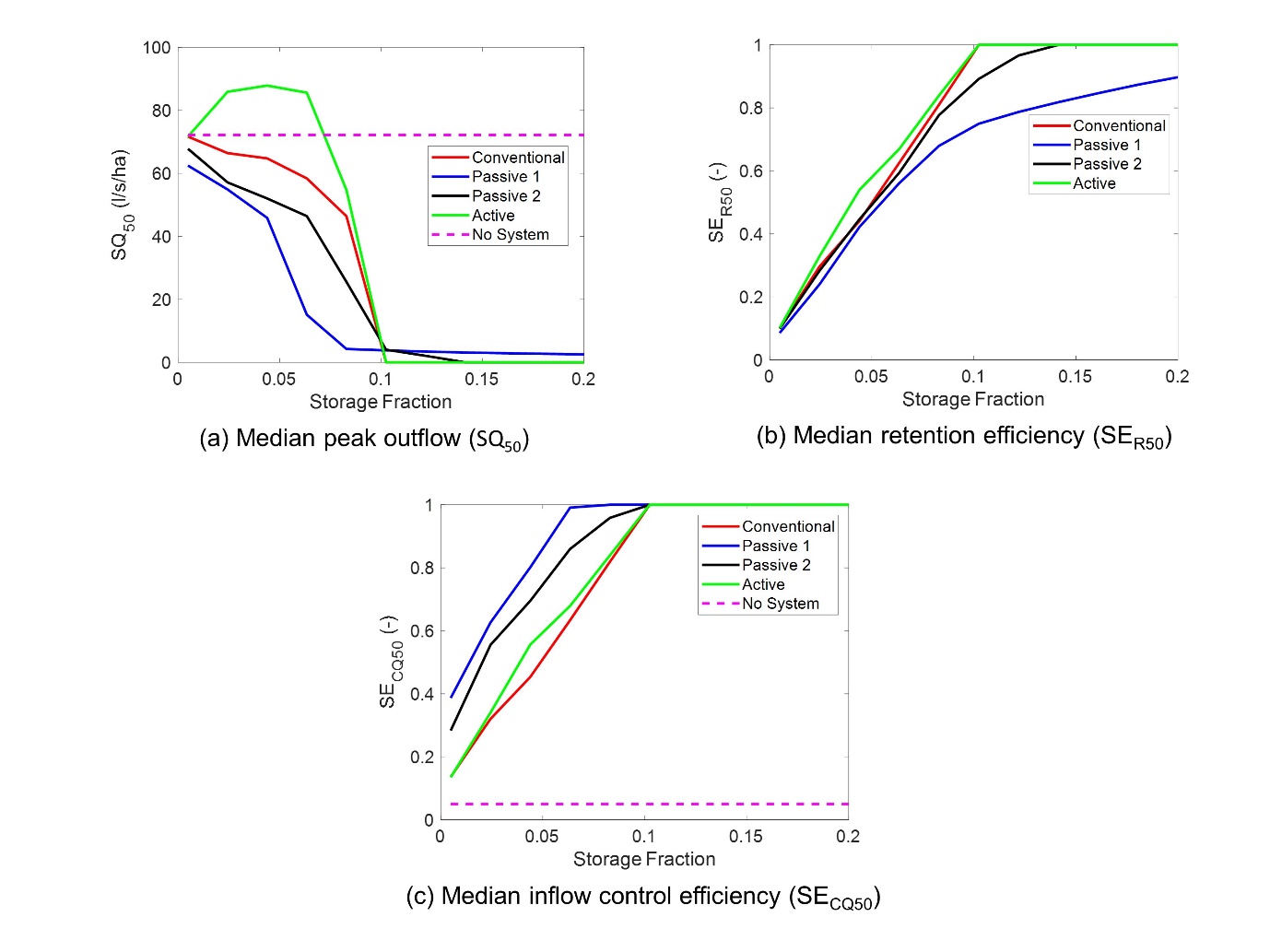


Storage Sensitivity Analysis for Events with the Largest 24h Rainfall Depth


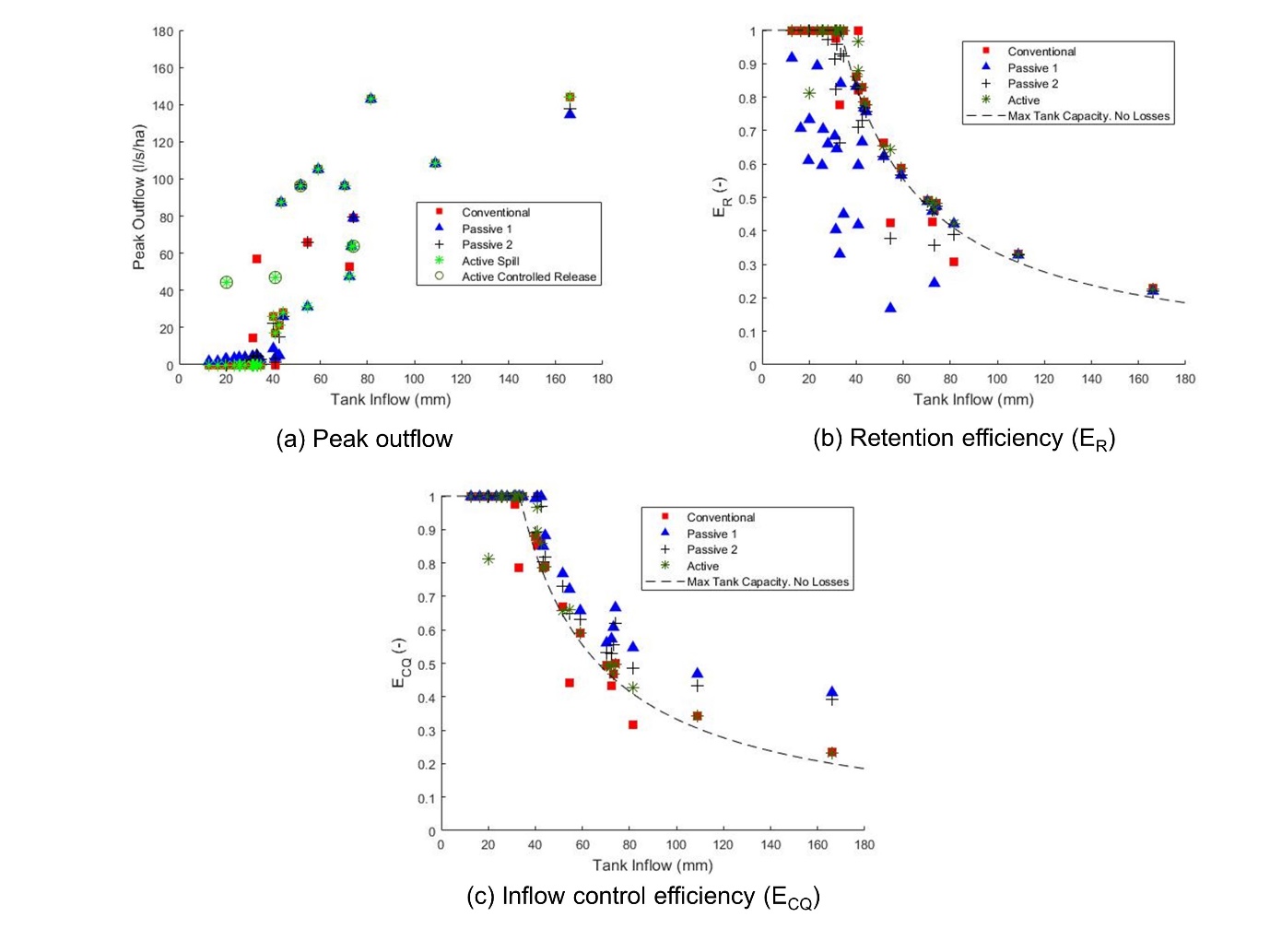


Scatterplots of System Performance during Events with the Largest 1h Rainfall Depth


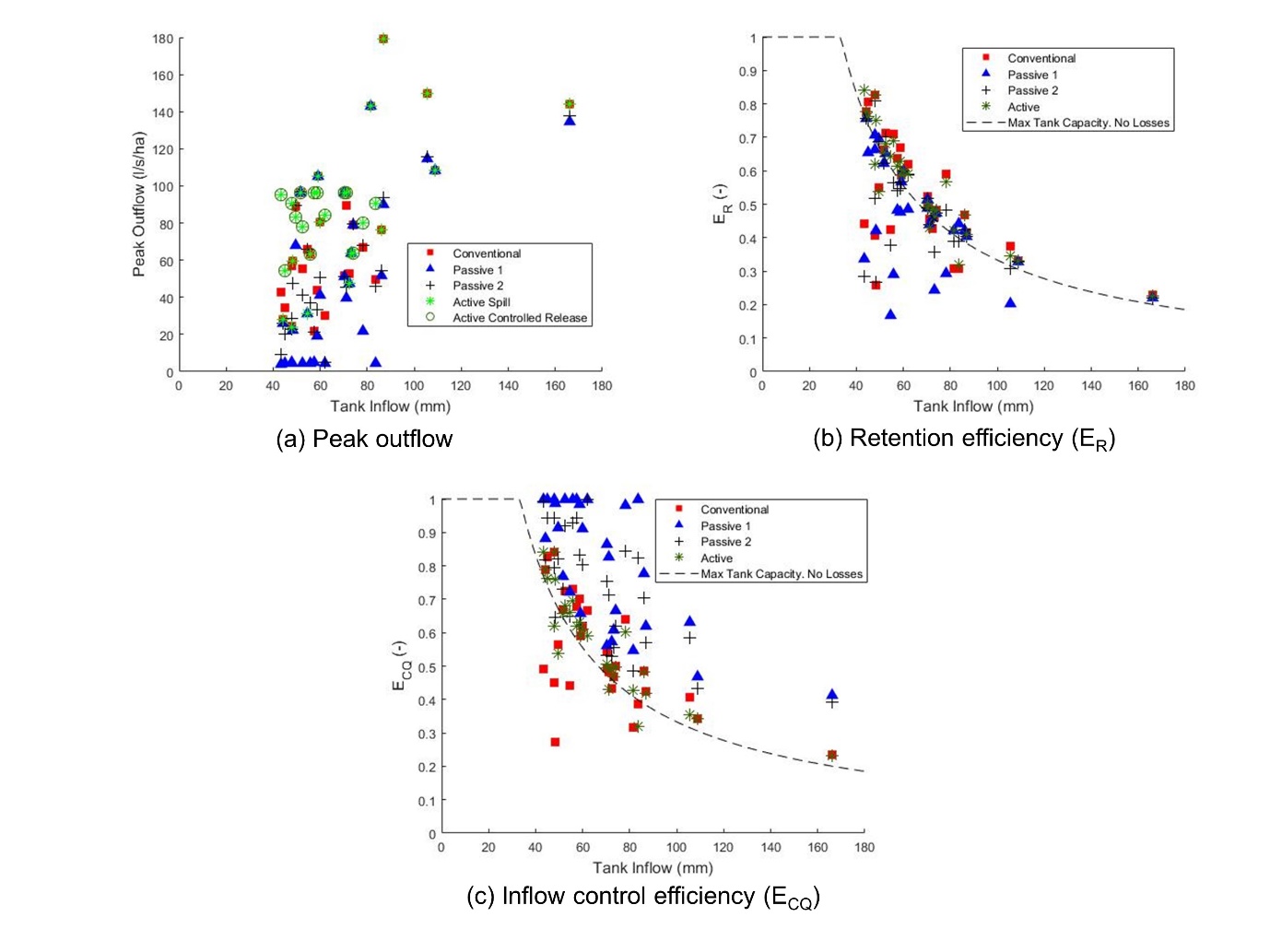


Scatterplots of System Performance during Events with the Largest 24h Rainfall Depth
